# Caregiving can be costly: A qualitative study of barriers and facilitators to conducting kangaroo mother care in a US tertiary hospital neonatal intensive care unit

**DOI:** 10.1186/s12884-019-2363-y

**Published:** 2019-07-04

**Authors:** Todd P. Lewis, Kathryn G. Andrews, Elyse Shenberger, Theresa S. Betancourt, Günther Fink, Sunita Pereira, Margaret McConnell

**Affiliations:** 1 Department of Global Health and Population, Harvard T.H. Chan School of Public Health, 677 Huntington Ave., Building 1, 11th Floor, Boston, MA 02115 USA; 2 University of Illinois at Chicago, Chicago, IL USA; 3 Boston College School of Social Work, Chestnut Hill, MA USA; 4 Swiss Tropical and Public Health Institute, Basel, Switzerland; 5 Tufts Medical Center, Boston, MA USA

**Keywords:** Preterm infant, Kangaroo mother care, Neonatal intensive care unit, Caregiving, Skin-to-skin contact, Qualitative methods, United States

## Abstract

**Background:**

Preterm birth is a leading cause of morbidity and mortality in children under five and often requires a newborn to have an extended stay in a neonatal intensive care unit (NICU). Maternal engagement, such as visiting the NICU to provide kangaroo mother care (KMC), can improve outcomes for preterm infants but requires significant investment of time and resources. This study sought to understand barriers and facilitators to provision of KMC in the NICU.

**Methods:**

We conducted semi-structured in-depth interviews with mothers of preterm infants (*N* = 20) at a large academic medical center in Massachusetts. A series of open-ended interview questions were designed to elicit all aspects of mothers’ experiences and to understand how these experiences influence provision of KMC. All interviews were recorded and transcribed verbatim. We conducted an inductive thematic analysis to identify themes in the data with a focus on the barriers and facilitators of KMC provision in the NICU.

**Results:**

Findings show that engaging in KMC is heavily influenced by the mental, emotional, and physical effects of preterm birth on the birth mother, such as stress around preterm birth and difficulty recovering from birth. These challenges are compounded by structural barriers such as costly accommodations, unreliable transportation, lack of child care, and inadequate maternity leave policies that limit the frequency and duration of KMC and parental ability to provide care.

**Conclusions:**

A complex array of mental, emotional, physical, and structural factors determine a mother’s ability to visit the NICU and provide kangaroo mother care. Providing social supports, such as improved maternity leave policies and reliable hospital access through child care, accommodation, and transportation services, may address the structural barriers that inhibit KMC, reduce burdensome costs, and improve the health of mothers and their preterm infants.

## Background

Preterm birth is the leading cause of death in children younger than 5 years of age worldwide [1]. In 2016, approximately one out of every 10 infants born in the United States was born premature [2]. Preterm infants, those born before 37 weeks of gestation, have higher risk of morbidity and developmental delays, as well as breathing problems, feeding difficulties, vision problems, and hearing impairment [3, 4]. Further, preterm birth is associated with developmental, cognitive, and behavioral problems in adolescents, and an increased risk of disease in adulthood [5–9]. Stark disparities in health outcomes of preterm infants persist along racial/ethnic and socioeconomic lines [10, 11]. For example, studies have shown associations between poorer socioeconomic condition and increased risk for preterm birth, as well as increased rates of preterm birth among black women even after accounting for socioeconomic factors [12, 13].

Kangaroo mother care (KMC), originally proposed as an alternative to conventional incubator care in resource-limited settings, is currently considered one of the most cost-effective interventions to promote the wellbeing of preterm infants [14, 15]. KMC involves three primary components: 1) skin-to-skin contact, 2) frequent and exclusive breast feeding, and 3) early discharge from the hospital [16]. KMC is typically initiated once an infant is stabilized, providing a source of nutrition, stimulation, and support to the infant while it matures. Skin-to-skin contact can stimulate breast milk supply, stabilize the infant’s heart rate, and improve the infant’s breathing pattern [17]. Further, KMC has been shown to improve thermoregulation and improve the infant’s behavioral state among other potential benefits [18], as well as facilitate a “bonding effect” between mother and child and a “resilience effect” in which women feel more competent as mothers [19, 20]. Research shows that KMC can mitigate the increased risks of morbidity and mortality among preterm infants [14].

Despite the documented benefits, coverage of KMC across hospitals in the United States is highly variable and a variety of barriers may inhibit mothers from practicing KMC. One survey of US neonatal intensive care units (NICUs) indicated that KMC was practiced in some form in 82% of all facilities and 67% of Level 3 NICUs nationwide, though updated estimates are needed [21]. While many hospitals support and actively promote KMC, some women face barriers to following recommended KMC practices [22]. One study found that mothers had insufficient time to conduct KMC given parental obligations, and that feeding-related activities such as breastfeeding and breast milk expression caused interruptions in skin-to-skin contact [23]. Another study identified stress and level of communication with the medical staff as key determinants of a mother’s ability to visit the NICU and engage in skin-to-skin contact [24]. Recent studies highlight barriers to implementing KMC such as insufficient time, social support, medical care, and family acceptance, as well as “resource-related” barriers such as issues with the facility environment. However, these studies focus primarily in low- and middle-income countries and largely assess the perspectives of clinicians rather than parents. None of the identified studies both solicited the perspectives of mothers and examined potential structural barriers to KMC within a US population [15, 22, 25].

Many studies have explored the negative mental and emotional aspects of preterm birth and the effects on parents [26, 27]. Parents face the shock of unexpected early birth, alienation due to the stress of the NICU experience, pressures of building a relationship with their infant, difficulties communicating with the neonatal care team, and struggles balancing new responsibilities [28–34]. However, less is known about barriers to engaging in KMC in the NICU such as the demand on mothers’ energy, time, and financial resources, or facilitators that may address those barriers; few studies have used in-depth interviewing to explore these factors, with many focused on low- and middle-income country contexts where hospital environments differ substantially in the services they provide to families to support KMC [22, 35–38]. We used Andersen’s Behavioral Model of Health Services Use to better understand barriers and facilitators to utilization of KMC in the inpatient setting in a high-income country context [39]. The results of this qualitative study will inform future work on facility-based interventions to address barriers to KMC and other forms of maternal caregiving in the NICU.

## Methods

### Setting and sample

This study took place in the NICU at Tufts Medical Center, a large academic medical center in downtown Boston, Massachusetts. The facility NICU, a Level 3 nursery in Tufts’ Floating Hospital for Children, receives referrals from community hospitals and affiliates throughout New England and often serves as a safety net for low-income families with preterm infants in need of higher level care. In 2016, approximately 49% of infants admitted to this NICU were covered by public insurance. The open bay facility contains two overnight rooms available to parents with infants in the NICU, small lounge areas for families, and armchairs for parents to sit at the bedside. Parental visitation is highly encouraged at any time through several organizational policies. A protocol to encourage KMC for the duration and frequency desired by parents has been in place in the NICU since 2010. Parents are encouraged to engage in KMC as much as possible once the child is deemed stable by the care team. Nurses frequently help situate parents and prepare them (e.g., adjusting clothing, positioning the child, etc.) for skin-to-skin contact with their infant.

Study participants included mothers of preterm infants who received or were receiving inpatient care at the Tufts Medical Center NICU (Table 1). We focused on mothers rather than partners or other family members as mothers were most likely to be present in the NICU and were the primary participant in certain aspects of KMC, such as breastfeeding; mothers will also be an important focus of future interventions planned by the research team to enable caregiving, including KMC, in the NICU and therefore are the primary population of interest. Interviews were conducted with 20 mothers (*N* = 20). Mothers ranged in age from 28 to 41, with an average age of 33 years. Their infants’ gestational ages ranged from approximately 30 to 37 weeks, with an average gestational age of 33 weeks. Just over half of mothers who could identify their health insurance provider had coverage through a state Medicaid program, while the remainder were privately insured. Approximately half of mothers reported living less than 1 hour from the NICU by the mother’s chosen mode of transportation, with an average distance of 52 minutes. Only 20% of mothers had any paid maternity leave.

**Table 1:** Description of mothers with preterm infants: participant-reported characteristics

Variable		# reporting (%) (*N* = 20)
Mother’s age		
	25–29	5 (25)
	30–34	8 (40)
	≥35	5 (25)
	Unknown	2 (10)
Child’s gestational age		
	30–32 weeks	9 (45)
	33–34 weeks	7 (35)
	≥35	3 (15)
	Unknown	1 (5)
Time to hospital		
	< 60 min	11 (55)
	≥60 min	9 (45)
Insurance provider		
	Public	13 (65)
	Private	6 (30)
	Unknown	1 (5)
Paid maternity leave		4 (20)
Mother had twins		4 (20)

### Study procedures and data collection

We used a qualitative descriptive design and an inductive thematic analysis approach based on semi-structured in-depth interviews with mothers of preterm infants in the NICU. Interviews were conducted by the second author, a doctoral candidate specializing in early life health and development, using an interview guide created by the author team and designed based on their subject matter knowledge and clinical expertise. The interviewer asked a standard set of questions across interviews, but allowed divergence from these questions based on interviewee responses. The interviewer probed mothers on their experiences having a preterm infant, their knowledge of and experience with kangaroo mother care, and perceived barriers and facilitators to engaging in skin-to-skin contact, breastfeeding, and breast pumping. We focus on skin-to-skin contact and breastfeeding and pumping, but not early discharge, as we expect these factors to be most affected by barriers and facilitators to caregiving during hospitalization. Interviews included open-ended questions such as “How do you decide when to come to the hospital to visit your baby?” Interview questions were crafted to elicit mental, emotional, and physical elements of mothers’ experiences and to identify any structural barriers, such as logistical or financial difficulties, that may have affected mothers’ ability to care for their children. While there was no quantitative survey component to the study, participants were asked a short set of limited demographic and logistical questions, allowing authors to assess certain self-reported characteristics such as mother’s age, insurance status, or distance from the hospital to provide context to the findings.

Criterion sampling, a form of purposeful sampling that aims to identify and select all cases that meet predetermined criteria of importance [40], was used to identify mothers eligible to be interviewed based on both mother and infant characteristics: Mothers had to be at least 18 years of age and able to speak and understand English or Spanish. Infants had to meet the following criteria: 1) currently a patient in the study NICU (either born in or transferred to the NICU for care of prematurity), 2) born between 30 0/7 and 36 6/7 weeks gestational age (when infants are stable enough to engage in KMC), and 3) spent at least 7 days in the NICU. Nurses helped to identify mothers whose infants were eligible for participation based on the infant’s charts, conversations with the mother, and discharge timing. Nurses and other NICU staff are intended to support the practice of KMC through educating mothers, enabling breastfeeding or pumping with the support of lactation consultants, and encouraging skin-to-skin contact whenever possible. Eligible mothers were asked by phone if they were willing to be interviewed about their experiences as a mother with a preterm infant. If they agreed, the study team member attempted to schedule an interview at the interviewee’s convenience at the hospital or at a regional facility if the child had already been transferred. Mothers received information about the study both verbally and in writing, were informed they could end the interview at any time for any reason without affecting their experience in the NICU, and were assured of data confidentiality. A study team member acquired verbal consent from participating mothers before each interview. Interviews were conducted in a private space (or semi-private space when necessary) in the hospital between September 2016 and January 2017. They were conducted in English or Spanish, audio-recorded, and lasted between 30 and 60 minutes. Researchers conducted interviews until theme saturation was reached. Theme saturation was defined as the point at which additional interviews did not lead to new emergent themes [40].

### Data management and analysis

All interviews were transcribed verbatim in Microsoft Word from audio-recordings. Interview recordings were transcribed by the lead author or transcribed and translated by another member of the study team if in Spanish. The resulting transcripts were de-identified, seen only by study team members, and housed securely in an online storage service. Detailed interview memos and field notes were reviewed continuously by the study team throughout data collection. Interview transcripts, also reviewed continuously through the interview period, were organized and analyzed to identify common themes regarding mothers’ experiences having a preterm infant and performing kangaroo mother care. Following an inductive thematic analysis approach [41], the first author read the transcripts repeatedly to become familiar with the data, developing initial codes of interest with no prior assumptions or guiding theory according to grounded theory techniques [42]. These codes were then categorized into broad categories and sub-categories and organized into a codebook to be applied to the entire dataset. The first author used a coding software, Dedoose (version 7.5.19), to help organize and support the coding process. The author applied the codebook in Dedoose to a sample of transcripts and iterated the codebook based on new codes emerging from the data. Emerging codes and their application to sample data were reviewed within the study team to improve reliability of application to full transcripts. The codebook was then applied to the entire dataset to identify key themes in the data, allowing for axial coding, including visual displays of the data, to identify co-occurring themes and better understand relationships between themes. We examined coded transcripts to identify the most commonly occurring themes and the importance mothers ascribed to said themes in terms of their own perceptions of how influential a given factor was to their NICU experience. We also report responses to a short set of demographic and logistical questions included in interviews, as well as the proportion of interviews in which a given theme was identified. To strengthen the validity of findings, we triangulated uncoded interviewer field notes and post-interview memos on contextual and interpersonal observations with themes identified in coded interview transcripts. Identified themes were also reviewed by subject matter experts on the author team, including a neonatologist and a NICU administrative staff member, to help contextualize the findings within the study setting.

We used Andersen’s Behavioral Model of Health Services Use to explore factors determining utilization of KMC in the NICU. The model’s three major components include predisposing factors, need factors, and enabling factors that can serve as barriers or facilitators to health and health services [39, 43]. In this case, predisposing factors include maternal factors, such as demographic or mental characteristics (e.g., stress), that determine whether a mother engages in KMC. Need factors include both perceived need (e.g., a mother’s own perception of KMC and its value) and evaluated need (e.g., prompting to engage in skin-to-skin contact by a clinician) for KMC. Enabling factors include organizational, institutional, and financial factors that determine NICU visitation and therefore the opportunity to conduct KMC, such as insurance coverage or travel time to care. Use of Andersen’s model allows us to situate themes within an existing theoretical framework of utilization, understand the dominant barriers and facilitators influencing mothers’ behaviors, and identify areas for future work.

## Results

**Fig. 1:**
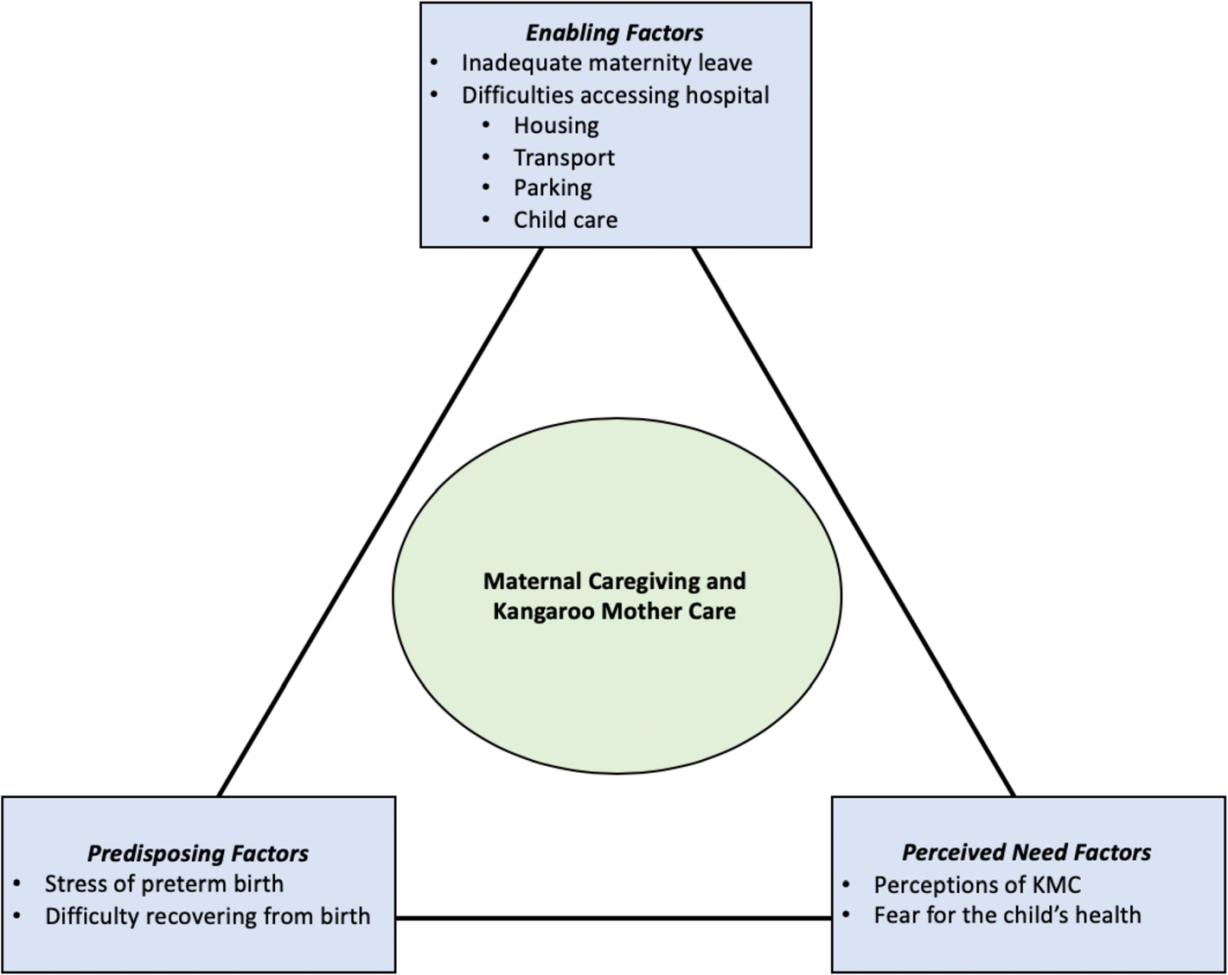
Predisposing, need, and enabling factors that influence maternal caregiving

Study findings suggest that the extent to which mothers can engage in KMC is determined by each type of factor in Andersen’s model (Table 2). Predisposing factors included barriers such as stress of preterm birth and difficulty recovering from birth. Need factors, all of which related to perceived need, included perceptions of KMC (a facilitator) and fear of impacting the child’s health (a barrier). Enabling factors included structural barriers such as a lack of maternity leave and difficulties accessing the hospital. Our findings indicate that ability to visit the NICU—which is required to engage in KMC—among financially-strained families is heavily influenced by these structural barriers and their associated costs, burdening all participating mothers regardless of hospital financial support or insurance status. Figure 1 provides a visual depiction of each of these themes organized within Andersen’s framework and we discuss each in detail below.

**Table 2:** Identified themes categorized by predisposing, need, and enabling factors

Theme	# interviews (%) (*N* = 20)
Predisposing Factors	
Stress of preterm birth	20 (100%)
Breast pumping discomfort/pain	6 (30%)
Breast pumping stress	11 (55%)
Difficulty recovering from birth	16 (80%)
Perceived Need Factors	
Perceptions of KMC	
Bonding	15 (75%)
Enjoyment	15 (75%)
Improved milk production	6 (30%)
Prompting by nurses	14 (70%)
Fear for child’s health	14 (70%)
Fear of making the child cold	6 (30%)
Fear of disturbing child/equipment	5 (25%)
Enabling Factors	
Inadequate maternity leave (i.e., too short or unavailable)	17 (85%)
Difficulties accessing the hospital	
Housing	13 (65%)
Transportation	17 (85%)
Parking	15 (75%)
Child care	9 (45%)

### Predisposing factors

#### Stress of preterm birth

Upon first giving birth, mothers reported initial shock and the feeling of being overwhelmed. They felt the process of unexpected hospitalization and sudden birth was “crazy” and happened quickly, one noting: “… the first few days, it was almost surreal. I couldn’t believe that I wasn’t pregnant, and, you know, had a baby.” Another commented, “It’s been tough. As, after all, it’s very unexpected. And … having to adjust to so many things at the same time … I was expecting to give birth normally …” Mothers suggested that spending time in the NICU after the shock of an early birth was emotionally and physically taxing: “It is hard. It’s emotionally draining … I hate the hospitals. I don’t like being—all these monitors are on … but I know what it means for [my daughter].” For many mothers, this feeling of shock was accompanied by complex feelings of having been “cheated” out of a full pregnancy and a feeling of guilt regarding their child’s health challenges: “I feel guilty as a mother to see him suffering and not be able to do anything about it. Sometimes you feel like it should have been you instead of him because he’s so little.”

Mothers also reported stress related to feeding their newborns by breast pumping or breastfeeding, especially in terms of producing enough milk and managing a pumping schedule. Over half described breast pumping as stressful, painful, uncomfortable, or taxing (55%). One mother commented: “Trying [to pump] now … it’s the most stressful thing.” Another noted that “Every time my alarm goes off on my phone … I look over at that thing [the pump] and I want to break it.” Others cited the rigorous schedule of breast pumping as prohibitive to engaging in more skin-to-skin contact. However, mothers cited nurse encouragement as an important facilitator impacting their decision to breastfeed: “At first I was not going to breastfeed and we were just going to do formula. But then the nurses were telling us all the benefits of pumping and the nutrition of the breastmilk, so then we were doing that.” As this mother indicated, nurse knowledge-sharing enabled some mothers to breastfeed more regularly while in the NICU.

#### Difficulty recovering from birth

In conjunction with these complex emotions, mothers’ caregiving was heavily influenced by their own recovery from birth. Many of the participants had caesarean sections, and/or gave birth in urgent or near-urgent contexts. One described the stress of the surgery: “From my room through delivery it was six minutes, the doctors just having to get me open.” Mothers reported feeling pain and discomfort related to this experience and pushing through the pain to spend time in the NICU to care for their new child. A participant noted: “Recovering from the C-section was awful … I couldn’t cough because I felt like my stomach was being ripped apart but … I still came down [to the NICU] the next day to see him.” They noted the discomfort of sitting in the NICU for long hours, attempting to perform usual maternal activities such as holding and feeding their child while enduring back and stomach pain, and forgoing sleep or meals to remain present with their infant. These aspects of physical recovery limited self-care and influenced mothers’ ability to devote energy to their child’s care.

### Perceived need

#### Perceptions of kangaroo mother care

Nineteen mothers reported engaging in skin-to-skin contact at least one time for several minutes or more, with nurses initiating the vast majority of skin-to-skin contact encounters (70%). Some mothers reported never having been offered to conduct skin-to-skin contact, and one reported having to request or suggest it herself. Mothers were generally enthusiastic about the practice, one noting: “Every time a nurse comes around and offers for us to hold her, we’re like ‘Yeah! I’m not saying no to that!’” Their KMC knowledge was primarily facilitated by nurses in the NICU, friends and family who had previous experiences with preterm birth, and internet sources. Approximately 30% of mothers reported having heard nothing about KMC from nurses, or not remembering how they learned about the practice. All 20 mothers reported having encountered the term “skin-to-skin care” or “kangaroo care” at some point.

Despite this familiarity, most mothers could only identify one to two benefits of KMC. Most frequently, mothers acknowledged bonding as a key benefit. One commented: “Kangaroo care is supposed to be beneficial to the mom and to the baby and I guess I can say from personal experience that you do feel like you get that sense of bonding and it’s so sweet.” A minority of mothers identified skin-to-skin contact benefits such as temperature regulation (20%) and breathing regulation (15%), and none mentioned breastfeeding or early discharge as beneficial components of KMC. Overall, mothers felt a strong sense of joy when holding their infants skin-to-skin, and perceived similar enjoyment in their child: “And then the feeling is like, you feel like you’ve never been in love until you met him. You know, that’s what I feel … It was like mommy and son time. You’ve never been in love until you met that little one.”

#### Fear of impacting the child’s health

Most mothers expressed fear for their child’s health, especially in terms of the infant’s size and ability to breathe. Mothers of children with severe health issues expressed deep, urgent concern: “I constantly worry: ‘Oh my God.’ Every day, like ‘Oh my God. Is she going to die because she is so little?’” This perception of the child’s health determined the mother’s caregiving behaviors, many fearing that activities like changing a child’s clothes or engaging in skin-to-skin contact might induce stress in their child: “I don’t want to stress her out and try to—I don’t want to advance her more than she needs to be right now,” potentially indicating a perception that “advancing” the child’s development through skin-to-skin contact might cause the child stress. Some mothers reported barriers such as a fear of making the child cold and stressing the infant too frequently. Less frequently, mothers were afraid that conducting skin-to-skin contact might harm the child due to his or her small size or that they might disturb the medical equipment, including “all the tubes” and “wires.” One mother reported that removing the infant from the incubator is “a big production” and that “you don’t want to stress [the infant] out.” For some, this fear inhibited caregiving activities, while for others their concern inspired more active monitoring of the child’s progress. Some mothers indicated they took every opportunity to “watch the numbers” (such as heart rate or oxygen saturation displayed on monitors), change diapers, assist with feedings, and alert nurses to any issues their child might be having.

### Enabling factors

#### Maternity leave

Mothers reported difficulties managing time in the NICU because of limited or inflexible maternity leave. While some reported flexibility in returning to their jobs, many left work earlier than planned: “Because I was getting so big … I started swelling, and back pains. A lot of aches. So I decided to stop, and since then I’ve not been back.” Most mothers received unpaid leave, with only four mothers reporting any paid maternity leave. Some mothers lacked maternity leave altogether and planned to re-apply to their job or seek a new job when returning to work. Many had to weigh taking time off from work to be present in the NICU against using their time off to care for the child after discharge. One mother described the dilemma: “I’m only getting paid once, so I’m either going two weeks unpaid, and then I’ll get paid whenever I’m with him being home, or I take my maternity leave now, but then I have no time for when he comes home.” For some, there was no debate—it would be impossible to layer work over obligations to “pump, see [my son], … and actually sleep.”

Few women reported being satisfied or feeling supported by their employers or maternity leave policies. For one mother, this had implications for her health: “… Part of the maternity leave isn’t just about the baby. It’s about you physically with all the pain and everything you went through, getting better … A lot of women go back [to work] sooner than that against the doctor’s wishes.” For others, this meant financial struggle and hard choices when living on one income (or on their savings) or forgoing adequate or affordable insurance. One mother commented: “Well, you have credit cards. You have bills. Insurance. Car insurance. Car payments, everything. So when there’s only one person working it’s not the same. It’s like you’re living paycheck to paycheck basically, so both times I got pregnant I lost my job, and then my bills keep going up and up and up, and I still can’t keep up with them.” The loss of a steady income during time spent in the NICU created additional stress for these mothers and presented a barrier to spending additional time in the hospital. One mother stated: “… if we could come more often, we could hold them more often. But it’s hard to come more often … with Christmas coming up and all the bills and this that and the other, we come as much as financially possible.”

#### Accessing the hospital

In addition to employment and leave struggles, mothers expended substantial resources to visit the NICU. Their main concern was accessing adequate and affordable accommodation near the NICU and managing transport from home to the hospital. Many families praised available “parent rooms” where families could live within the hospital adjacent to the NICU for free during their infant’s time as an inpatient. This facilitated visitation, eased the effort required to travel, and relieved financial burden on parents. For mothers who were not able to access a room, the experience was taxing: “The first night I cried when I left because they didn’t have any rooms available and I didn’t want to leave her.” Some of these parents managed a local hotel stay at significant cost, but for others, this was prohibitively expensive even with a hospital discount.

Mothers also reported challenges related to the distance to the NICU and coordinating transportation by car or train. The community hospital close to home may not have had a NICU or may not have been equipped to handle a high-risk pregnancy, requiring transfer to the larger referral hospital where they delivered. Post discharge, most mothers were not able to drive per medical recommendation and reported difficulties scheduling their visits around family obligations, train schedules (or the schedules of family/friends offering to drive them), or their infant’s feeding times. Both car and train were identified as expensive modes of travel in terms of fuel and fare, though hospital-provided gas cards ($50.00 each) eased some of this burden. For those that drove to the hospital, the long distance (ranging from 10 minutes to 3 hours depending on traffic) and the cost of parking were considered burdensome even with discounted parking vouchers provided by the hospital for hospital parking facilities.

Outside the hospital, many parents commented on the challenge of balancing time spent in the NICU with their obligations to their other children. Stress, parental obligations, and difficulty scheduling time in the NICU were commonly co-occurring sentiments. One mother commented: “I wish I was here more often, but like I said, when you have somebody else depending on you, you can’t be in two places at the same time.” Another mother, considering her struggles to access the NICU, noted: “You just adjust … You don’t think about the barriers. You just do what you have to do.” Forced absence from their other children created an additional stressor and logistical barrier for these mothers.

Despite these barriers, mothers reported feeling supported by hospital social workers. Social workers provided financial resources, such as parking vouchers and gas cards, and mental/emotional resources such as parental support groups. One mother commented: “[The social worker] checks on us if we need anything, if we need parking vouchers, if we have any questions. We also have her [contact] card so we know we can always call her. She’s been awesome.” In many cases, mothers reported they would not have known about resources available from the hospital were it not for the efforts of the social workers, and suggested that social workers played an important role facilitating financial supports.

## Discussion

The findings of this study provide a rich perspective on the key characteristics of mothers’ experiences in the NICU and barriers and facilitators to providing KMC after a preterm birth. A primary contribution is that visiting the NICU, one with active supports for KMC and an existing KMC protocol, to engage in KMC is inhibited by complex structural barriers including insufficient maternity leave and challenges accessing the referral hospital in terms of accommodations, transportation, and child care. These challenges are associated with high costs for families and persisted across participants regardless of self-reported external financial supports, mother’s insurance status, or other facilitators. Our findings suggest that these structural barriers impact a mother’s ability to visit the NICU and engage in KMC. Better understanding of these barriers and how they may affect financially-strained families, including many in this study who cited costs as a significant problem, is essential for building a comprehensive model of child health that accounts for a fuller range of social and environmental factors [44].

### Predisposing factors

Findings confirm previous literature suggesting that an array of predisposing factors related to a mother’s mental and physical health can permeate the NICU experience, including stress, under-preparedness for the newborn, difficulties coordinating visits and feedings, and other NICU-related obligations [26, 28, 33]. Mothers’ comments indicated negative feelings, anger, and fatigue, associated in other literature with elevated rates of psychological distress [26, 27, 31, 32]. These emotions led some mothers to seek greater involvement in their child’s care [28, 33, 45, 46].

Expanding on previous literature, our findings suggest that a mother’s physical recovery from birth greatly impacts her NICU experience in terms of both her willingness to be in the hospital and her ability to engage in her child’s care. Activities such as sitting to provide skin-to-skin contact for multiple hours were a painful ordeal, and spaces for rest and relaxation were not always available in the NICU. Mothers reported ignoring their own basic needs in deference to the needs of their children, forgoing meals and rest to continue watching over or spending time with them. Mothers may benefit from support from family and health providers to perform self-care, both to improve their own health and to safeguard their ability to care for their infants. In addition, new models of parental involvement, such as family-integrated care models that enable parents to become primary caregivers in the NICU, have shown positive mental and physical effects for both infants and parents and may be an important step forward in neonatal care [47].

Despite physical challenges, our findings highlight positive perceptions of KMC as a key facilitator. Mothers and their children achieved strong enjoyment and bonding from KMC, and skin-to-skin contact in particular. This feeling of bonding was a central predisposing factor in mothers choosing to conduct skin-to-skin contact in the NICU and continuing to conduct it throughout the stay. In contrast to past work, mothers did not explicitly identify feelings of alienation, struggles to bond, or challenges associated with becoming a mother [29, 30, 48]. In fact, many mothers in this study actively sought opportunities to bond with their newborns through skin-to-skin contact. This difference may be due in part to the health of these infants, who were robust enough to be safely held, and also due to the existence of a KMC protocol in the NICU which may have made nurses more comfortable in encouraging mothers to engage in this activity. Regardless, capitalizing on this positive sensation of bonding may help facilitate engagement in skin-to-skin contact within the NICU.

Our study findings also indicated that breast pumping and breastfeeding were highly stressful for mothers in terms of the physical experience of regularly expressing milk and the coordination involved with mothers’ pumping schedules. Access to high quality pumps and insurance coverage of pumps for home-use were crucial to enable mothers to provide expressed breast milk for their preterm infants who could not effectively suckle; nearly every mother’s breast pump was covered by her insurance, reducing costs for these mothers and encouraging breast pumping. As seen in previous studies, support from the NICU nursing staff and lactation consultants was instrumental [30, 49, 50]. Health providers should consider bundling skin-to-skin contact and breast pumping under the KMC umbrella to routinize their use and capitalize on the joint benefits of these practices.

### Perceived need

While mothers reported positive feelings from engaging in KMC, they also reported knowing very little about the full range of its benefits and were concerned that engaging in skin-to-skin contact or breastfeeding might disturb or harm their child. As found in previous work, nurses played an essential role in increasing the prevalence of KMC, engaging mothers in its practice, and educating them about its importance [21, 51]. Nurse encouragement around KMC was often the first time mothers had learned about skin-to-skin contact, and mothers suggested they may never have requested to conduct it without prompting by nurses. Further, nurses served to assuage fears and dispel common misconceptions about skin-to-skin contact, such as the risk of making the infant cold or of disturbing the medical equipment. However, these infants are typically stable enough to engage in skin-to-skin contact, and parents were encouraged by nurses to perform skin-to-skin despite the presence of intimidating medical equipment. Nurses played an essential role in overcoming these fears, alerting parents to their child’s needs, and facilitating KMC while in the NICU.

### Enabling factors

A primary contribution of our study is the importance of enabling factors to the NICU experience for interviewed mothers. Mothers faced numerous structural barriers such as inadequate maternity leave policies and difficulties accessing the hospital. These findings are particularly stark given Massachusetts’s relatively substantial social safety net and robust Medicaid program. In 2017, the Commonwealth Fund ranked the Massachusetts state health system fifth in the country across 40 measures of access, quality, cost, and equity [52]. Despite this, mothers repeatedly identified these structural barriers and their financial consequences as central determinants of their experiences. Existing studies that examine structural barriers primarily feature supply-side barriers, such as inadequate facilities or poor communication among clinical staff, and focus on the experience of conducting KMC in low- and middle-income countries [22]. Further, we identified no US-based studies that examined the out-of-pocket costs mothers face and their ramifications for the NICU experience. Our findings suggest that these enabling factors determine both a mother’s own recovery and her ability to invest in her child’s health while in the NICU. Eliminating structural barriers may have direct benefits in terms of visiting the hospital, but may also be required for addressing aforementioned predisposing and need factors such as reducing stress or enabling maternal self-care.

One chief determinant of mothers’ experiences was maternity leave, a feature often excluded from similar studies conducted outside the US in settings where paid maternity leave is commonplace. Mothers reported struggling to support themselves and their families without a steady income. In some cases, partners (particularly those working hourly wage jobs) who wished to spend time in the NICU or to drive a mother to the hospital were not able to work as many hours, placing additional constraints on family income. This led to hard choices and additional stress for some mothers, who had to choose between being present in the hospital to care for their child and paying monthly bills. The central challenge of inadequate parental leave underscores many of the other logistical challenges these mothers face. In Massachusetts, state law requires employers with six or more employees to provide 8 weeks of unpaid parental leave to both men and women [53]. While this policy is generous compared to other US states, it was insufficient to safeguard the mothers who participated in this study. This study highlights the need for parental leave policies that take into consideration the particular challenges faced by families with preterm infants, who may spend weeks in the hospital and require additional adjustment time after discharge.

New legislation in Massachusetts taking effect in 2019 will make employees eligible for paid parental leave, including partial wage replacement and up to 12 weeks to care for a newborn (50% longer than the current leave duration), extendable to 26 weeks for addressing medical complications from pregnancy, birth, or postpartum recovery. The legislation would also prohibit employer retaliation for those that take family leave under these conditions. Such laws could help mothers maintain their positions during pregnancy, ensure regular income during the NICU experience, provide additional leave for adjustment after hospital discharge, and guarantee the mother’s job upon her return. These protections may be particularly impactful for low-income families, whose children are more likely to be preterm and who may struggle to support themselves during their infant’s time in the NICU.

Our results also highlight the importance of affordable accommodations during an infant’s time in the NICU, especially given general financial demands of the NICU experience and the high cost of hotels in an urban center. Mothers who could not stay in or near the NICU noted the emotional toll of not having immediate access to their children. A similar study showed this burden was relieved by having constant access to the NICU, day or night, either in person or by phone [45]. However, consistent with prior evidence, NICU caregiving was facilitated by nearby accommodations: mothers were most at ease, both emotionally and financially, when they had access to the hospital’s limited overnight rooms in or near the NICU [23].

Other financial burdens associated with accessing the hospital included transportation and parking. Parents spent significant time traveling to the hospital, often while juggling a job, other children, and a taxing breast pumping schedule. Coordinating these activities was inconvenient and uncomfortable for mothers, especially those recovering from physical trauma from birth. Mothers were also constrained by their inability to drive post-surgery and found that the public transportation schedules were too restrictive to be a viable mode of transport. Though mothers benefited from facilitators such as hospital-provided gas cards and train fare, these supports could not cover all travel-related expenses. Many parents noted that parking, either on the street or in the hospital garage, became cost prohibitive for long stays.

Stakeholders in the health of mothers and children, such as policymakers, insurers, and hospital systems, should emphasize new ways to support mothers by focusing on these structural challenges. For example, hospitals could explore the provision of social supports, such as overnight living spaces or onsite child care, to alleviate the logistical burdens on mothers. Further, providing supports to families could facilitate visitation and skin-to-skin contact by partners, an area for future research. At a state level, longer, paid maternity leave policies should be tailored to the unique needs and burdens faced by mothers with preterm infants [54]. Our study also highlights the beneficial role of social workers for parents of preterm infants. Recent guidelines for social workers in the NICU have focused largely on addressing maternal and paternal mental health challenges. However, social workers can serve as a first line of defense in tackling structural barriers and facilitating caregiving [54, 55]. Expanding the role of social workers to address a range of logistical challenges may be a valuable policy tool. Without interventions to address these barriers, preterm infants, especially those from low-income families, may not reap the benefits of parental investments in KMC, which could exacerbate disparities and limit infant health and survival.

### Limitations

Some study limitations should be noted. While every effort was made to interview mothers in private locations, the presence of family members or hospital staff was occasionally required. This could impact whether mothers were able to share their opinions and experiences freely. In addition, as clinicians encourage mothers to engage in KMC, mothers may have felt pressure to report these activities, especially while physically present in the NICU. Tufts Medical Center, our study hospital, is highly supportive of KMC, has a standard protocol for KMC, and actively promotes it among patients. However, hospital policy regarding skin-to-skin contact, breastfeeding, or NICU visitation will vary by hospital system; in some hospitals KMC may not be a formalized practice or discussed with parents at all. Further, these findings represent the experiences of mothers receiving care at one large academic medical center in Massachusetts, a state with a strong social safety net, and may not reflect the experience at all hospitals or of all mothers with preterm infants. Finally, in terms of study sample, while the number of participants may be considered low, thematic saturation was reached very early on, and did not require additional interviews. However, we were limited in our ability to disaggregate findings by certain important characteristics. In particular, exploration among racial/ethnic minorities who may either directly experience other important barriers, including racism or discrimination, or who may have limited trust in health care providers because of prior related experiences, is necessary to obtain a more nuanced view of structural barriers within the context of existing disparities. [56, 57] It is important to note that these findings are exploratory, not exhaustive, and there may be other characteristics of the NICU experience not captured in this study. Despite these threats to validity, the themes were common across the multiple forms of data analyzed. Themes emerged from initial inductive analysis, but were also identified through triangulation across multiple qualitative media, including interviewer field notes and post-interview memos.

## Conclusions

This study is among the first in-depth analyses of how predisposing, need, and enabling factors influence KMC utilization among US mothers with preterm infants. Our findings indicate that this experience is characterized by a complex array of barriers and facilitators that determine a mother’s ability to visit the NICU and provide KMC. To improve the NICU experience for mothers and promote the health of preterm infants, social supports, such as improved maternity leave policies and reliable hospital access through child care, accommodation, and transportation supports are required, even for parents with insurance coverage. Addressing these factors through policy changes and hospital interventions is essential to enabling optimal maternal caregiving while an infant is in the NICU. Further research is needed to identify scalable solutions that address the emotional, physical, and structural barriers these mothers face, and to ensure the health of both mother and child.

## Data Availability

The data that support the findings of this study are available on reasonable request from the corresponding author [TPL]. The data are not publicly available due to them containing information that could compromise research participant privacy.

## Abbreviations

KMC: Kangaroo mother care
NICU: Neonatal intensive care unit
